# Individual Response to Stressors and Efficiency in Project Activities

**DOI:** 10.3390/bs10010010

**Published:** 2019-12-20

**Authors:** Yulia A. Dmitrieva, Svetlana Yu. Korobova, Darya V. Kochkina

**Affiliations:** 1 Department of Psychology of Development and Age Consultation, Department of Psychology, Higher Medical and Biological School, South Ural State University, 454080 Chelyabinsk, Russia; 2 Laboratory of Psychology and Psychophysiology of Stress-Resistance and Creativity, Scientific and Educational Center “Biomedical Technologies”, Higher Medical and Biological School, South Ural State University, 454080 Chelyabinsk, Russia; korobovasi@susu.ru; 3 Foreign Languages Department, Institute of Linguistics and International Communications, South Ural State University, 454080 Chelyabinsk, Russia; kochkinadv@susu.ru

**Keywords:** stress, stressors, project activity, project-based learning, efficiency of project activity, individual responding, human resources, cerebral hemispheres activation

## Abstract

This article investigates the way a graded approach can be implemented in the organization of the project-based learning process in accordance with the personality characteristics of subjects. This study is based on the results analysis of the key features of project-based learning as one of the methods for developing human resources. The assessment of subjects’ individual response to stressors at different project stages is justified to be relevant in order to improve project efficiency in the framework of the learning process. The assessment of the individual response of subjects to stressors was carried out according to the activation dynamics of cerebral hemispheres. The research target was to determine features of the subjects’ individual responses to the project activity stressors and to identify specifics of the individual responding at each of its stages. The study involved 56 final-year students of different majors. *Aktivatsiometr ATs-9K*, a device for comprehensive psychophysiological diagnosis designed by Yu. A. Tsagarelli, was used to monitor hemispheric activation. This hardware and software complex consists of a device diagnosing the activation of cerebral hemispheres based on the galvanic skin response and PC software for automatic data processing. The individual typological indicators of activation (13 measurements in a familiar environment) and activity-situational indicators of activation (seven single measurements at different stages of the project activity) were calculated. The stress response was diagnosed if the activity-situational activation indicators of the cerebral hemispheres exceeded the individual typological activation indices by more than 1.5 times. The results of the empirical study show different types of individual responses to stressors at different stages of the project activity. The analysis of the profiles of individual responses to stressors made it possible to differentiate subjects, and also identify groups of students with the maximum resources for each stage of activity.

## 1. Introduction

Project activity is becoming one of the dominating forms of human activity organization and is implemented in a number of spheres: (1) administrative, as one of the dominating forms of organization management in the present-day social and economic conditions; (2) educational, as one of the efficient learning methods in pre-school [1] and school educational institutions, and in the arrangement of work in higher education institutions [2,3,4]; and (3) research, as a way of planning and organizing scientific studies [5]. In each of these spheres, project activity has both common patterns and features, and its own specifics. 

Project activity spread is due to the presence of a particular activity structure via the designation of specific stages with special conditions for their implementation and the possibility of controlling the efficiency of such activity at every stage.

This study aims to search for a differentiated approach to project activity organization and its effectiveness enhancement in terms of the subjects’ personal characteristics. Thus, based on the project activity features considered above, it is topical to examine the specifics of the individual responding to the main stressors of the project activity at its various stages.

The research target is to determine features of the subjects’ individual responding to the project activity stressors and to identify specifics of the individual responding at each of its stages.

The research hypothesis is that the efficiency of the project activity at various stages is determined by the subjects’ individual responding to the stressors, which manifests itself in the specifics of the cerebral hemisphere activation dynamics.

## 2. Literature Review

It is necessary to organize continuous preparation for the conditions and requirements of project activity from preschool educational institutions to higher education institutions. Project activity is the best way to prepare for the real conditions of organizations’ functioning within the scope of human potential development [6]. 

The organization of project activity is better implemented in higher education institutions, which provide not only professional knowledge and competencies, but so-called soft-skills as well. The term “soft-skills” has a number of meanings and is generally defined by researchers as the combination of certain qualities and skills of a person [7,8,9]. Soft skills are synonymous with transferable skills and the opposite to hard-skills [7]. 

Researchers distinguish general and specific qualities that relate to soft skills. The majority of studies reveal that critical thinking, teamwork, and communication skills relate to general qualities, whereas the ability to work in a stressful and uncertain environment relate to specific qualities [8]. 

It has also been proven that the development of soft skills allows graduates to be better adapted to the real conditions of professional activity [9]. 

Most studies also reflect the general structure, main stages, and principles that should be followed when implementing a project-based learning process [10,11,12].

Thus, J. Garcia-Martin and J.E. Perez-Martinez analyzed the problematic area of project-based learning and detected the main stages and principles [10].

J.R. Mergendoller and J.W. Thomas theoretically substantiated three main phases of project-based learning: definition, support, and organization [11]. R.J. DeFillippi defined basic principles of project-based learning through the analysis of their practical application. These principles include: time management, getting started, establishing a culture that stresses student self-management, managing student groups, working with others outside the classroom, getting the most out of technological resources, and assessing students and evaluating projects [12].

In addition to the above, individual factors affecting the efficiency of project-based learning are studied. The research by M.K. Noordin and M.S. Nordin determined three main factors that contribute to the development of team-working skills. These skills include supervision, evaluation throughout the entire project realization, and the presence of real global experience associated with the project subject [13].

Project-based learning is characterized by a number of positive achievements (obtaining soft-skills, including communication skills, leadership, and teamwork skills), and has certain disadvantages (high level of stress generation, tension and uncertainty of the activity).

On the one hand, implementing this form of work in learning activities has some undeniable advantages. A number of authors have pointed out that the overall efficiency of the learning activity increases and the level of spiritual and moral upbringing grows [14]. Thus, students advance their creative and intellectual activity [15], increase motivation [16], and develop communication skills with their peers as well as with their teachers. 

On the other hand, project activity, as a special form of the subjects’ interaction, has a high level of stress generation [17]. The analysis of various stages and requirements of the project activity reveals the main stressors, i.e., a high level of tension and uncertainty, the need to organize mutual activities and make group decisions, and the evaluation of professional competences. Project activity tension is associated with the pre-determined execution deadlines and the team-working nature of the activity, including that in the heterogeneous groups [18,19]. The uncertainty of such activity is determined by a great number of possible strategies of the project realization and the need to make both individual and group decisions. Professional evaluation of the subjects’ activity relates to the daily stress problems. The way such problems are dealt with, individual qualities, and age-related specifics of the subjects become especially important in this case [20,21,22,23,24,25].

All the above-mentioned factors highlight the problem of students’ stress resistance and adaptation in critical, uncertain, and tense situations, in combination with maintaining or even increasing the efficiency level of their activities [26,27]. 

The effectiveness of project activities and the success of a particular subject in such activities depend on a multitude of parameters, such as the imposed requirements and activity implementation conditions [28], as well as individual characteristics of the participants [29].

## 3. Materials and Methods 

### 3.1. Research Design

An experimental synthetic model that combines fundamental and practice-oriented studies was implemented in this project [29]. The determination of general psychological patterns of the cerebral hemispheres’ activation dynamics was supplemented by a differential psychological approach to the studies of the subjects’ individual differences. 

The experimental research model included equipment-based psychophysiological diagnostics of the students’ individual responding to the stressors during three main stages of the project-based learning activity.

Stage 1: The students were supposed to determine the subject matter of their academic projects, the level of complexity, creativity, and novelty, supervision independence (with or without their tutor’s supervision), the project submission deadline (within or before the timelines). For this purpose, a questionnaire form was designed containing the above-listed parameters to determine the project implementation conditions, namely the project subject matter, submission deadline, the number of consultations with the supervisor, the complexity of the selected subject matter, and compliance with the layout standards. There were suggested options for each parameter ranking: selection of one option or the other set the total score range and made it possible to qualify for a certain grade (final grade of the subject learning).

Stage 2: The students presented their academic project with the visual presentation of the key outcomes. Each student had to present the results of their academic project to a committee consisting of a number of teachers acting as experts. The specific stressors at this stage of the project activity were the necessity of public speaking in the context of professional evaluation situation, uncertainty of the ultimate outcome, and complexity of communication establishment.

Stage 3: This stage of the project activity included discussion of the results of the academic project presentations and announcement of the final grade of the subject learning. The main stress factor of this stage was the uncertainty of the ultimate activity outcome due to the existing multitude of evaluated parameters.

All three stages were carried out within one semester, successful subject mastery and passing the subject examination were considered to be the outcome of the academic project realization.

The efficiency of each student in the project activity (project implementation) was evaluated within the range of 40 to 100 points. The subject matter side of each project was evaluated by expert practitioners specializing in the respective subject area. 

To ensure the most trustworthy and reliable results in the research of the project activity efficiency, the method of contrasting groups was employed—that is, differentiation of groups of students who possessed the maximum and minimum efficiency in the project activity. 

All subjects gave their informed consent for inclusion before they participated in the study. The study was conducted in accordance with the Declaration of Helsinki, and the protocol was approved by the Ethics Committee, protocol number is 307/20 of 5 December 2019.

### 3.2. Research Sampling

The study involved 56 senior students, aged 22.5 on average. There were 21 male and 35 female participants. Three groups of students of different majors (technical, economic, and social humanities) were chosen due to the practice of project activity implementation into the educational process. The sample scope was not divided by majors, since the purpose of the study was to investigate the general patterns of individual responding to stressors in project activities without taking into account the specifics of the students’ majors.

To implement the method of contrasting groups when comparing specifics of the individual responding to the stressors, the entire sample scope was divided into five groups, in accordance with the project performance level. The average value of the project activity efficiency (xav = 72 points) and the standard deviation (σ = 18 points) were calculated in this respect.

- group 1 (n_1_ = 8 persons)—students with the highest project performance, above 91 points;
- group 2 (n_2_ = 17 persons)—students with a good level of project performance, above the norm, over 81 points;
- group 3 (n_3_ = 12 persons)—students with normal project performance (64 to 80 points);
- group 4 (n_4_ = 8 persons)—students whose project performance is below the norm, below 63 points;
- group 5 (n_5_ = 11 persons)—students with the lowest project performance, below 54 points.

In accordance with the method of contrasting groups, for further study of the specifics of individual responding to the stress factors of the project activity, group 3, with normal project performance, was excluded from the comparison. The psychological diagnostics data of groups 1, 2, 4, and 5 were used in the comparison.

### 3.3. Research Methods

The assessment of the individual response of subjects to the stressors of project activities was carried out on the basis of changes in cerebral hemisphere activation. To study the dynamics of the cerebral hemisphere activation at various stages of the project activity, the *Aktivatsiometer ATs-9K* was employed. This is a hardware–software complex for psychophysical diagnostics by Yu. A. Tsagarelli. The method of cerebral hemisphere activation diagnostics was based on the registration of the galvanic skin reflex (GSR) on the palms [30]. GSR registration made it possible to determine with high accuracy the level of cognitive load experienced by a person, as well as the level of their emotional reaction to it [31].

Both individual and typological, and activity and contextual indices of the cerebral hemisphere activation were used at various stages of the project activity.

Individual and typological indices of the cerebral activation were calculated according to 13 measurements in a customary, comfortable environment (x_av_ = 103, σ = 63). Activity and contextual indices of the cerebral hemisphere activation were obtained by single measurements at various stages of the project activity (before, during, and after determination of the project execution conditions; before and after presentation of the academic project outcomes; before and after the announcement of the project presentation results)—seven measurements in total.

The stress response was diagnosed if the activity and contextual characteristic of the cerebral hemisphere activation exceeded the individual and typological characteristic of the activation more than 1.5 times.

Experimental research data of the subjects’ individual responding to the stressors and analysis of the theoretical and empirical studies made it possible to determine three types of the cerebral hemisphere activation dynamics:

1. lack of significant (more than 1.5 times) changes in the aggregate activation of the cerebral hemispheres—normal responding, no stress or shock response;
2. increase in the aggregate activation of more than 1.5 times compared to the individual and typological characteristic—stress response;
3. decrease in the aggregate activation of more than 1.5 times compared to the individual and typological characteristic—shock response.

## 4. Results

The frequency distribution of various types of cerebral hemisphere activation dynamics in each student group was compared. The importance of the differences in terms of the number of stress and shock responses in the student groups was evaluated using criterion χ2 and binominal m-criterion. Results are given in Table 1 below.

When comparing the student groups differentiated by their project activity efficiency (criterion χ2), statistically significant differences in the number of stress and shock responses were determined. 

Student group 2, whose efficiency in the project activity was above the average, demonstrated a significantly greater number of stress responses as compared to the students of other groups under the project activity conditions. Students with efficiency levels above the average in the project activity, once affected by the stress factors of the project activity, more frequently experienced stress as a response of a dramatic increase in the cerebral hemisphere activation and mobilization of all resources.

The students of group 2, with above-average efficiency, and the students of group 5, with minimal efficiency of the project activity, were characterized by a significantly greater number of shock responses compared to the students from other groups. 

When comparing the number of stress and shock responses in the student groups differentiated by their project activity efficiency (m-criterion), the statistically significant differences were determined. 

Student groups 1 and 5, which had the maximum and minimum indices of the project activity efficiency, typically demonstrated a predominance of the number of shock responses over stress ones. Thus, both the most efficient and the least efficient students much more frequently experienced shock as a response to reduction in the cerebral hemisphere activation under the impact of stress factors of the project activity.

Subsequently, the specifics of individual responding to the stress factors of the project activity at its various stages were analyzed. The importance of the differences in terms of the number of both stress and shock responses in the student groups at various stages of the project activity was evaluated using criterion χ2. The results are given in Table 2 and Table 3.

Significant differences were determined in the number of stress responses among the student groups differentiated by efficiency at various stages of the project activity. At stages 1 and 2 of the project activity, stress responses were found much more frequently in student group 2 compared to the students from other groups. Thus, students whose project activity efficiency is above average are the most liable to have stress responses at the stages of determining the project activity conditions and public presentation of the projects.

Significant differences were revealed in the frequency of shock responses at various stages of the project activity in the student groups where project activity efficiency was above and below the average. 

The students of group 2, with above-average efficiency, typically experienced a significant predominance of shock responses at stages 1 and 3 of the project. These stages were characterized by a high level of uncertainty. The students of group 4, with below-average efficiency, predominantly had shock responses at stage 1 of the project activity and no shock responses at stage 2. Thus, the shock responses prevailed at this stage, which included a high level of uncertainty and making group decisions. The stage including the situation of public evaluation caused no shock responses.

## 5. Discussion

An experimental design scheme of project-based learning was formulated in accordance with the basic principles and phases presented in the works of J. Garcia-Martin, J.E. Perez-Martinez J.R. Mergendoller, J.W. Thomas, R.J. DeFillippi, K.F. Gray, and E.U. Larson [10,11,12,32].

An analysis of the research showed that there are several stages of project-based learning. The most common stages of project activities are the goal setting stage, the stage of direct project implementation, and the stage of presenting project results and their evaluation. These three phases of project-based learning are consistent with numerous studies on project management, studying the project life cycle, and using project activities in an educational institution [32].

Thus, the first stage of project-based learning, which includes the selection of the theme and conditions of the project, corresponds to the phases of discussion and organization by J.R. Mergendoller and J.W. Thomas [11]. The principles of time management and creating culture, which emphasize student self-government, by R.J. DeFillippi, are also realized within this framework [12]. The second and third stages of experimental project-based learning correspond to the phases of organization and support according to J.R. Mergendoller and J.W. Thomas. The principle of project appraisal is implemented in the framework of the second stage, the principle of student assessment by R.J. DeFillippi can be found in the third stage [11,12].

Project-based learning, as well as other types of academic activity, makes students have a stress reaction [27,33]. It was revealed that the greatest number of stress reactions in the framework of the experiment was demonstrated by students with a project performance efficiency higher than average. Also, within all stages of project training for all groups of students, there is a predominance of the reaction to reduce brain activation, shock. This can be interpreted as a decrease in adaptive activity to new, uncertain conditions of activity.

Analysis of changes in brain activation at different stages of project-based learning revealed that the first stage is the most challenging one. This stage includes choosing the topic and conditions of the project, and it causes the greatest number of reactions to increase and decrease brain activation. This stage, in accordance with the principles of time management and creating a culture of student self-government, requires a high level of self-organization and self-regulation from students. This is consistent with the studies of N.H. Hj Ramli, M. Alavi, S.A. Mehrinezhad, and A. Ahmadi [33], confirming the relationship of academic stress with a low level of student self-regulation.

## 6. Conclusions

Three types of responses to the stressors in the project activity were revealed: a stress response, a shock response, and retention of the cerebral activation level within the individual and typological norm. 

At each stage, groups of subjects with different types of responding can be differentiated.

Subjects with above-average efficiency of the project activity demonstrated the presence of a stress response under the project activity conditions more frequently than others. The most stressful stages for them were those associated with target setting and selection of the project activity conditions, as well as the public situation of evaluation of the project activity results.

Subjects with both above-average and below-average efficiency of the project activity demonstrated a great number of shock responses related to a significant decrease in the cerebral activation at the stage associated with target setting and selection of the project activity conditions. 

The limitations of this study are primarily associated with the sample scope, which was not very large, and biased in majors. In the future, the sample scope of the study can be expanded by enlarging the total number of students of various majors. 

Hardware diagnostics of the activation of cerebral hemispheres based on the galvanic skin reflex on the palms sets certain limitations as well. The reliability of the research results may be improved by the simultaneous combination of hardware diagnostics and various tests of psychological diagnostics and other methods of psychophysiological research. 

Future perspectives of this study may include having a similar study of individual responding to stressors in project activities in the context of real-life activities and production process. Also, one of the options for further research is to study the possibility of predicting the effectiveness of project activities based on individual responding to stressors with the application of mathematical model methods. 

## Figures and Tables

**Table 1:** Comparison of the types of cerebral hemisphere activation dynamics in the student groups, differentiated by their project activity efficiency.

Student Group No.	Project Activity Efficiency	Number of Stress Responses	Number of Shock Responses	M-Criterion
1	maximum	1	12	0.01
2	above norm	30	32	-
4	below norm	5	12	-
5	minimal	6	27	0.1
χ2		41.02 **	11.67 **	

Designations: ** *p* ≤ 0.01.

**Table 2:** Comparison of the number of stress responses in the student groups at various stages of the project activity.

Student Group No.	Project Activity Efficiency	Stage 1	Stage 2	Stage 3	χ2
1	maximum	0	0	1	2.0
2	above norm	8	14	8	2.4
4	below norm	0	2	3	2.8
5	minimal	0	4	2	4.0
χ2		22.0 **	18.2 **	6.5	

Designations: ** *p* ≤ 0.01

**Table 3:** Comparison of the number of shock responses in the student groups at various stages of the project activity.

Student Group No.	Project Activity Efficiency	Stage 1	Stage 2	Stage 3	χ2
1	maximum	7	2	3	3.5
2	above norm	17	4	11	7.94 *
4	below norm	8	0	4	8.0 *
5	minimal	14	4	9	5.56
χ2		4.24	4.30	4.55	

Designations: * *p* ≤ 0.05.

## References

[1] Veraksa A.N., Veraksa N.E. (2008). Organization of project activities in kindergarten. Sovrem. Doshkol’noye Obraz. Teor. I Prakt..

[2] Dvoretskiy S., Puchkov N., Muratova E. (2003). Forming of a project culture. Higher Ed. Russia.

[3] Antyukhov A.V. (2010). Project-based learning in higher school: Problems and perspectives. Higher Ed. Russia.

[4] Emel’yanova N.V. (2011). Project activity of students in the educational process. Higher Ed. Today.

[5] Tregubov V.N. (2016). Features and prospects of using the project approach to ensure the innovation activities of the University. Innov. Deyatel’nost’.

[6] Bogomolova O.V. (2017). Project activities in the professional training of students. Vestn. Nauchnykh Konf..

[7] Andrews J., Higson H. (2008). Graduate Employability, ‘Soft Skills’ Versus ‘Hard’Business Knowledge: A European Study. High. Educ. Eur..

[8] Robles M. (2012). Executive Perceptions of the Top 10 Soft Skills Needed in Today’s Workplace. Bus. Commun. Q..

[9] Schulz B. (2008). The Importance of Soft Skills: Education beyond academic knowledge. J. Lang. Commun..

[10] Garcia-Martin J., Perez-Martinez J.E. (2017). Method to Guide the Design of Project Based Learning Activities Based on Educational Theories. Int. J. Eng. Educ..

[11] Mergendoller J.R., Thomas J.W. (2001). Managing project based learning: Principles from the field. Buck Inst. Educ..

[12] DeFillippi R.J. (2001). Introduction: Project-based learning, reflective practices and learning. Manag. Learn..

[13] Nordin M.S., Noordin M.K. (2018). Project-Based Learning (PjBL) Framework in Developing Non-Technical Skills for Engineering Students. Adv. Sci. let..

[14] Maksyutina A.I. (2017). Spiritual and moral education through project activities. Технoлoгическoе oбразoвание в шкoле и ВУЗе.

[15] Popova M.S. (2017). Project activities as a means of developing the creative activity of university students. Gaudeamus.

[16] CHusavitina G.N., Kurzayeva L.V. (2016). Experience in organizing project activities of students in the implementation of the master’s program “Information Technologies in Education”. Sovrem. Probl. Nauk. I obraz..

[17] Barabanshchikova V.V., Kaminskaya E.O. (2013). The phenomenon of procrastination in the activities of members of virtual project groups. Natsional’nyy psikhologicheskiy zhurnal.

[18] Fisher G. (2005). Developing social creativity: Let all voices be heard. Psikhologiya. ZHurnal Vyss. Shkoly Ekon..

[19] ZHuravlev A.L., Nestik T.A. (2010). Co-creation: State and prospects of research. Psikhologiya Intellekta I Tvorchestva: Traditsii I Innovatsii.

[20] Vasilyuk F.E. (1984). Psychology of Experience.

[21] Bodrov V.A. (2006). Psychological Stress: Development and Overcoming.

[22] Zankovskiy A.N. (1991). Occupational stress and functional states. Psikhologicheskiye Problemy Professional’noy Deyatel’nosti.

[23] Vodop’yanova N.E. (2009). Stress Psychodiagnosis.

[24] ZHuravlev A.L., Sergiyenko E.A., Tarabrina N.V., KHarlamenkova N.E. (2016). Modern aspects of the study of everyday and traumatic stress. Psikhologiya Povsednevnogo I Travmaticheskogo Stressa: Ugrozy, Posledstviya I Sovladaniye.

[25] Sushko N.G. (1998). Personality Determinants of Organizational Stress. Ph.D. Thesis.

[26] Price J.A., Morris Z.A., Costello S. (2018). The Application of Adaptive Behaviour Models: A Systematic Review. Behav. Sci..

[27] Iorga M., Dondas C., Zugun-Eloae C. (2018). Depressed as Freshmen, Stressed as Seniors: The Relationship between Depression, Perceived Stress and Academic Results among Medical Students. Behav. Sci..

[28] Likhacheva E.V., Ognev A.S. (2015). Key defects in education as factors of unpreparedness for project management. Mezhdunarodnyy zhurnal eksperimental’nogo obraz.

[29] ZHuravlev A.L., Ushakov D.V. (2011). Fundamental psychology and practice: Problems and tendencies of interaction. Psikhologicheskiy zhurnal.

[30] TSagarelli Y.A. (2009). System diagnostics of the person and development of mental functions: Textbook.

[31] Nourbakhsh N., Chen F., Wang Y., Calvo R. (2017). Detecting Users’ Cognitive Load by Galvanic Skin Response with Affective Interference. ACM trans. Interact. Intel. Syst..

[32] Grey K.F., Larson E.U. (2003). Project Management: A Practical Guide.

[33] Hj Ramli N.H., Alavi M., Mehrinezhad S.A., Ahmadi A. (2018). Academic Stress and Self-Regulation among University Students in Malaysia: Mediator Role of Mindfulness. Behav. Sci..

